# *Evx2*-*Hoxd13* Intergenic Region Restricts Enhancer Association to *Hoxd13* Promoter

**DOI:** 10.1371/journal.pone.0000175

**Published:** 2007-01-24

**Authors:** Takumi Yamagishi, Michiru Ozawa, Chiho Ohtsuka, Ritsuko Ohyama-Goto, Takashi Kondo

**Affiliations:** Kondo Research Unit, Brain Development Research Group, Brain Science Institute, Institute of Physical and Chemical Research (RIKEN), Wako, Japan; Baylor College of Medicine, United States of America

## Abstract

Expression of *Hox* genes is tightly regulated in spatial and temporal domains. *Evx2* is located next to *Hoxd13* within 8 kb on the opposite DNA strand. Early in development, the pattern of *Hoxd13* expression resembles that of *Evx2* in limb and genital buds. After 10 dpc, however, *Evx2* begins to be expressed in CNS as well. We analyzed the region responsible for these differences using ES cell techniques, and found that the intergenic region between *Evx2* and *Hoxd13* behaves as a boundary element that functions differentially in space and time, specifically in the development of limbs, genital bud, and brain. This boundary element comprises a large sequence spanning several kilobases that can be divided into at least two units: a constitutive boundary element, which blocks transcription regulatory influences from the chromosomal environment, and a regulatory element, which controls the function of the constitutive boundary element in time and space.

## Introduction

Protein-DNA interactions on DNA *cis*-regulatory elements and cross-talk between these complexes underlie precise transcription regulation. Since genes are embedded in huge DNA molecules containing abundant putative *cis*-regulatory elements such as enhancer and promoter sequences, the interactions of *cis*-regulatory sequences is extremely complicated. To achieve proper transcription regulation, selection of these interactions is inevitable. Some DNA regions are believed to play roles controlling traffic interactions among *cis*-regulatory elements.

Boundary elements divide a chromosome into independent units for transcription regulation. Mainly through genetic studies of *Drosophila melanogaster*, several candidate boundary elements (also known as insulator sequences or insulators) have been isolated [1]–[5]. Many of these elements act as components for homeotic gene regulation [1], [2], [4], [6], [7]. Enhancer activity must be well organized to achieve the proper transcription regulation of clustered homeotic genes needed for the development of proper anteroposterior segmental identity within an organism's body. Mutations within such boundary elements alter gene expression profiles by leading misuse of these enhancer sequences and cause morphological shifts in segmental identities [2], [8].

A similar phenomenon has been observed in mammalian orthologous homeotic complex genes [9], [10]. *Hox* genes are responsible for the anterior-posterior identity of the mammalian body, as is in the case of *Drosophila*. Misregulation of *Hox* genes causes morphological alterations [11]–[13] and can even be detrimental [14], [15]. As with *Drosophila*, enhancer-promoter interactions in mammals also require precise organization for *Hox* gene expression regulation [9], [10]. An enhancer that drives *Hoxd11* in the cecum cannot associate with the promoter of *Hoxd13*, which is about 10 kb away from *Hoxd11* [9], [10]. Intergenic deletion of* Hoxd12-Hoxd13*, however, causes *Hoxd13* to be expressed in the cecum in an expression pattern resembling that of *Hoxd11* [9], [10]. Taken together, these results indicate that the *Hoxd12-Hoxd13* intergenic sequence functions as an insulator which prevents enhancer access to promoter.

Here, we report another candidate insulator sequence in the *HoxD* complex—the *Evx2-Hoxd13* intergenic region. We demonstrated that this fragment possesses position-effect protection as well as insulator activity, two activities that are required for a sequence to be considered as a boundary sequence. Furthermore, we found that this boundary sequence functions in a tissue-specific manner, and that the regulation of tissue specificity can be separated from the boundary activity.

## Results

### Differential expression profile of *Evx2* and *Hoxd13*

**Figure 1:**
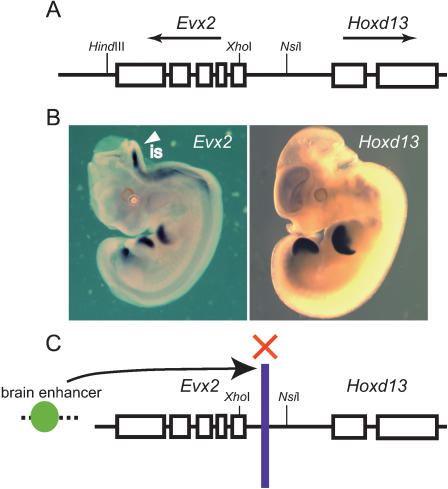
Genomic structure and expression patterns of *Evx2-Hoxd13.* (A) *Evx2* and *Hoxd13* genes are located within 8 kb of each other. (B) Expression patterns of *Evx2* (left panel) and *Hoxd13* (right panel) in 11 dpc embryos. *Evx2* is expressed in the CNS, as well as in the limbs and genital buds, regions that also express *Hoxd13.* (c) Scheme illustrating the hypothesis that *Evx2* and *Hoxd13* display segregated expression patterns. Enhancers located 3′ from *Evx2* have differential access to the *Evx2* promoter and the *Hoxd13* promoter. The intergenic region of *Evx2*-*Hoxd13* prevents enhancer access in CNS but not in limbs.

*Hoxd13* is located within 8 kb of the *Evx2* gene, which is encoded on the opposite strand of DNA from other *HoxD* genes (Figure 1A). Examination of 11 days post-coitus (dpc) embryos revealed that the expression profile of *Evx2* is distinct from that of its neighbor, *Hoxd13* (Figure 1B) [16]. In younger embryos, however, the expression profiles of *Evx2* and *Hoxd13* are almost identical. Expression of *Evx2* in central nervous system (CNS) begins at 10 dpc, being especially prominent in the isthmus (Figure 1B). *Hoxd13,* however, is never expressed in anterior structures throughout embryonic and fetal development (Figure 1B). We previously created mice harboring a series of *Hoxd9*/lacZ marker transgene insertions into the region surrounding *Evx2* (Figure S1A) [14]. When the transgene was positioned immediately downstream of the *Evx2* poly(A)^+^ signal (*rel*I), lacZ expression mirrored *Evx2* expression; however, when the transgene was positioned half-way between the *Evx2* and *Hoxd13* initiation codons (*rel*O), lacZ expression resembled *Hoxd13* (i.e., lacked CNS expression in 11 dpc embryos) (Figure S1B) [14]. Enhancer activities driving the expression of these genes in CNS, digits, and genital bud are located about 250 kb downstream from *Evx2* [17]. Taken together, these results suggest that an enhancer blocker element (insulator) exists in the vicinity of the *Evx2* promoter, and that this insulator prevents enhancer(s) from interacting with *Hoxd13* in the CNS (Figure 1C) [14], [18].

### Molecular dissection of *Evx2*-*Hoxd13* insulator activity

**Figure 2:**
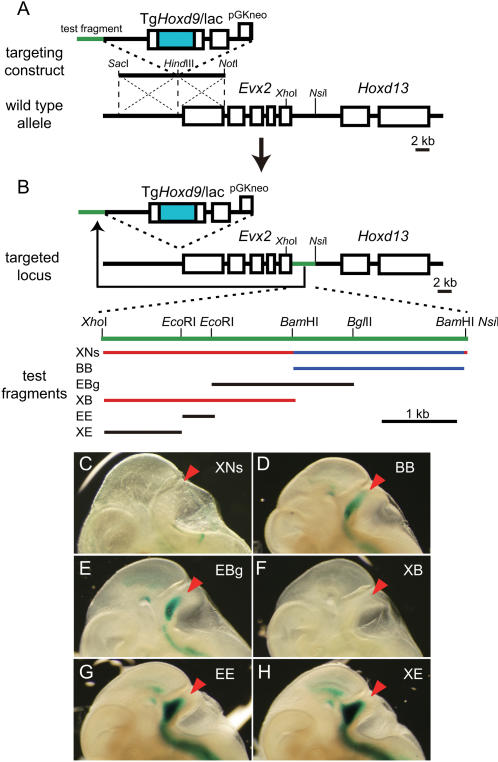
Analysis of the insulator fragment using targeted transgene experiments. (A) Scheme of experimental design. The *Hoxd9*/lacZ transgene, along with an insulator candidate fragment, was inserted into the region just downstream of *Evx2* by a gene-targeting technique using ES cells. The resulting ES cells were injected into blastocysts to establish transgenic mice. (B) The *Xho*I-*Nsi*I (XNs) fragment (red) was separated into three fragments—*Bam*HI-*Bam*HI (BB), *Eco*RI-*Bgl*II (EBg), and *Xho*I-*Bam*HI (XB)—each of which were translocated together with the *Hoxd9*/lacZ reporter transgene. (C–H) LacZ-stained 11 dpc embryos. XNs (C) and XB (F) blocked lacZ gene expression in brain, while BB (D) and EBg (E) failed to do so. Based on these results, we divided the XB fragment into two fragments—*Xho*I-*Eco*RI (XE) and *Eco*RI-*Eco*RI (EE)—which we used to make targeted transgenic mice having a similar configuration to that shown in panel (A). Embryos harboring XE (G) and EE (H) displayed lacZ gene expression in brain but did not display expression patterns indicative of insulation activity.

Using homologous recombination in ES cells, we translocated candidate boundary sequences to the region immediately downstream of *Evx2* along with the *Hoxd9*/lacZ reporter transgene (Figure 1A; Figure 2A, B). The resulting ES cells were injected into blastocysts to create transgenic mouse embryos. Chimeras and progeny from the chimeras were then stained for β-galactosidase activity. If the candidate boundary sequence has insulator activity, then lacZ expression pattern should resemble that of *Hoxd13*; otherwise, lacZ expression pattern should resemble that of *Evx2* (i.e., in the CNS).

From our previous observations [14], we predicted that the boundary sequence is located between the *Evx2* promoter and the *Nsi*I site, half-way between *Evx2* and *Hoxd13* (i.e., the *rel*O transgene insertion site) [14] (Figure S1). Indeed, when the entire candidate sequence and reporter transgene were inserted into the *rel*O transgene insertion site (i.e., the 5 kb fragment between *Xho*I site in exon1 of *Evx2* and *Nsi*I site), lacZ-staining pattern in embryos was similar to that of *Hoxd13*, indicating that the *Xho*I-*Nsi*I (XNs) fragment blocked enhancer activity in CNS (Figure 2C). This observation guided our experimental design.

To assess the insulator activity in more detail, we divided the XNs fragment into three overlapping segments of about 2 kb each—*Xho*I-*Bam*HI (XB), *Eco*RI-*Bgl*II (EBg), and *Bam*HI-*Bam*HI (BB)—starting from the region adjacent to the *Evx2* initiation codon (Figure 2B). We then prepared transgenic mice harboring each fragment along with the *Hoxd9*/lacZ reporter transgene. Transgenic animals harboring either EBg or BB displayed an *Evx2*-like expression pattern (Figure 2D, E), indicating that these two fragments failed to block interaction between the CNS enhancer and the *Hoxd9*/lacZ promoter (Figure 2). On the other hand, transgenic animals harboring XB displayed no transgene expression in the CNS, indicating that this fragment has insulator activity (Figure 2F; Figure S2).

To assess the insulator activity of the XB fragment further, we divided the 2.5 kb fragment into three pieces (Figure 2B) and found that none of these shorter fragments showed insulator activity by blocking enhancer interactions with the reporter transgene (Figure 2G, H). This indicates that a particular length of DNA containing multiple protein binding sites dispersed within the 2.5 kb XB fragment is required for proper insulator function and that insulator activity is a consequence of complex DNA-protein interactions (Figure 2). Bell and colleagues reported that the binding sequence of the transcription factor CTCF is necessary and sufficient for enhancer insulation activity of HS4 of the chicken β-globin locus [19]. However, unlike in the case of the β-globin insulator, we were unable to isolate a small DNA element that functioned as an enhancer blocker in our study system. In addition, an *in silico* search using TESS (http://www.cbil.upenn.edu/tess/) failed to identify any candidate association sites for CTCF.

### Neighboring sequence regulates *Evx2*-*Hoxd13* insulator activity

**Figure 3:**
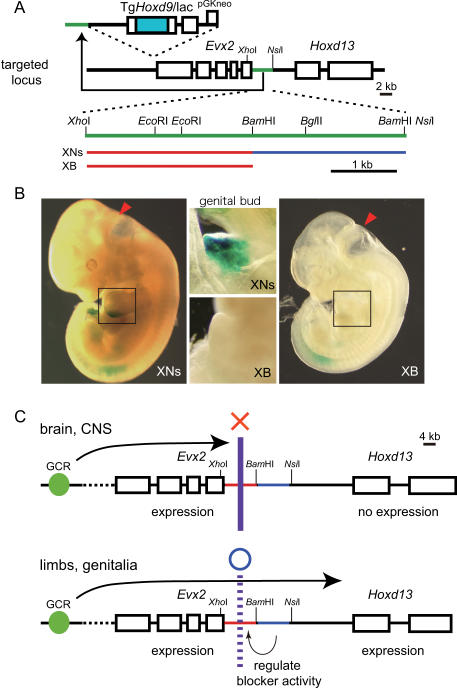
Insulation activity is spatially dependent. (A) Design of two targeted transgenic mice. XNs and XB as in Figure 2A. (B) Expression pattern of XNs- and XB-transgenic mouse embryos. XNs 11 dpc embryos expressed lacZ in the limbs and genital bud, whereas XB embryos did not. Limbs are shown in the boxed areas and genital buds are shown in the middle panels. (C) Scheme illustrating the regulation underlying enhancer-promoter interaction. Within the CNS, the XB fragment prevents interaction between the enhancer and *Hoxd13* promoter, while the BB fragment blocks the insulator activity of the XB fragment within the limbs and genital bud.

Comparison of the lacZ expression patterns of XNs- and XB-targeted transgenic mice revealed distinct differences (Figure 3A, B). As shown in Figure 2, both fragments blocked the expression of the lacZ reporter gene in the CNS. In XNs mice, we observed lacZ expression in the limbs and genital bud (i.e., resembling *Hoxd13* expression), whereas in XB mice, lacZ expression was absent in the limbs and genital bud (Figure 3B). These results suggest that (1) the XB fragment is a constitutive insulator, and (2) the specificity of the blocker activity is determined by a sequence in the *Bam*HI-*Nsi*I site (BNs) outside of the core blocker sequence, *Xho*I-*Bam*HI (Figure S3). The sequence in the BNs fragment may counteract or cancel the blocking activity of the XB fragment in limbs and genital bud, therefore, posterior *HoxD* genes are expressed in the limbs and genital bud (Figure 3C). Thus, the blocking sequence in the *Evx2*-*Hoxd13* system can be divided into two units—a constitutive blocker and a blocker regulator.

### Methylation status of *Evx2-Hoxd13* insulator sequence

To gain insight into the molecular mechanisms of the insulator activity of the XB fragment, we next analyzed chromatin structures for the methylation status of DNA residues, a key means of regulating gene transcription. Indeed, methylation is thought to contribute to gene silencing, as evidenced by the high frequency of methylated residues often observed in DNA surrounding silenced promoters [20]. It is probable that the methylation status of residues within functional insulator differs among organs or differs depending on a gene's transcription status, as observed in the case of the *H19* imprinting system [21], [22]. In our system, since insulation takes place only in hindbrain and not in limbs, we examined and compared the methylation status of DNA samples from the hindbrain and forelimb of 11 dpc embryos. The samples spanned over 1,700 bp of DNA fragment within the insulator fragment, as assessed by the bisulfite method [23].

**Figure 4:**
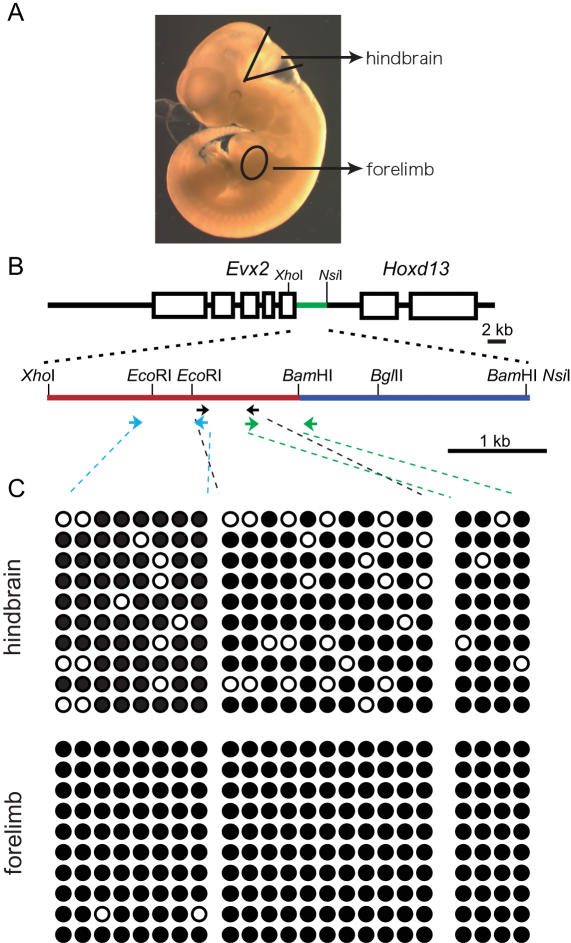
Methylation profile of insulator fragment. (A) Hindbrain and forelimbs were dissected from 11 dpc embryos to obtain the DNA used for the methylation assays. (B) Map of the tested fragment. Red line represents the insulator (XB fragment) and blue line represents the regulator (BB fragment). Bisulfite-treated genomic DNA was subjected to PCR using three sets of primers (indicated by arrows; see Experimental Procedures). Primer pairs for one PCR reaction have matching arrow color. (C) Ten clones from each PCR product were sequenced. Their methylation status is shown here. White circles represent methylated cytosine residues, while black circles represent non-methylated cytosine residues.

Hindbrain DNA samples contained methylated cytosine residues at relatively low to moderate frequencies (Figure 4). By contrast, forelimb DNA samples contained essentially no methylated residues (Figure 4), which is consistent with the absence of insulator activity in limbs.

### Lethality of targeted transgenic mice and *Hoxd13* expression

To further investigate the enhancer blocking activity of the XB fragment, we examined the expression patterns of *Hoxd13* and *Evx2* in targeted transgenic mice harboring the XB fragment. Our examination of several litters of 11.5 dpc embryos identified no homozygotic (XB/XB) targeted transgenic mice. With further analysis, we could not find the homozygous allele, even among 7.5 dpc embryos (Table 1). We did find, however, the BB/BB allele among 8.5 and 11.5 dpc embryos from one of the control lines harboring the BB targeted transgene (Table 1). Since lethality could not be segregated after more than 5 generations of out-breeding, we concluded that the lethal phenotype is closely linked to the presence of an additional copy of the XB fragment next to the *Evx2* gene.

**Table 1:**
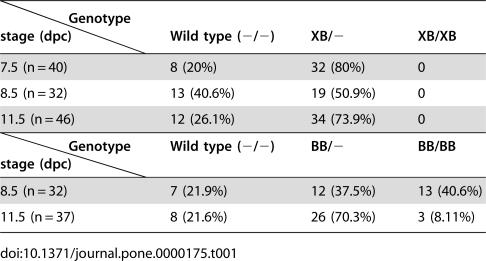
Segregation of genotype in internally bred, targeted transgenic mice

Genotype stage (dpc)	Wild type (−/−)	XB/−	XB/XB
7.5 (n = 40)	8 (20%)	32 (80%)	0
8.5 (n = 32)	13 (40.6%)	19 (50.9%)	0
11.5 (n = 46)	12 (26.1%)	34 (73.9%)	0
**Genotype stage (dpc)**	**Wild type (−/−)**	**BB/−**	**BB/BB**
8.5 (n = 32)	7 (21.9%)	12 (37.5%)	13 (40.6%)
11.5 (n = 37)	8 (21.6%)	26 (70.3%)	3 (8.11%)

**Figure 5:**
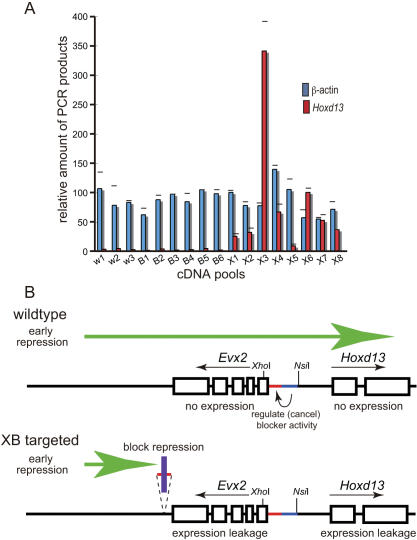
Premature expression of *Hoxd13* observed in XB-targeted transgenic mice. (A) *Hoxd13* expression in 7 dpc embryos was specifically upregulated. Sample RNA levels were normalized according to β-actin mRNA levels. W1–W3, samples from three litters arising from wild-type mice; B1–B6, samples from six litters arising from internally bred BB-transgenic mice; X1–X8, samples from eight litters arising from internally bred XB-transgenic mice. (B) Hypothetical scheme for premature expression of these genes in XB-transgenic mice. The region downstream of *Evx2* recruits repression over the *Hox* complex before the 7-dpc embryonic stage in preparation for *Hox* expression in wild-type and BB-targeted transgenic mice. The XB fragment disrupts repression, preventing the repressor region downstream of *Evx2* from recruiting repression into the *HoxD* complex.

To investigate the possible misregulation of *Hoxd13* or *Evx2* transcription resulting from insertion of the XB-containing transgene, we assessed *Hoxd13* and *Evx2* expression in 7 dpc embryos from the XB transgenic line by real-time PCR (Figure 5A). Three separate samples of cDNA prepared from three litters of internally bred wild-type mouse embryos and six samples of cDNA from six litters of internally bred BB mouse embryos were used as controls. We compared expression data from these controls to the *Hoxd13* expression measured from eight cDNA samples prepared from 8 litters of embryos resulting from the breeding of XB heterozygote mice. While litters resulting from the breeding of wild-type and BB mice did not express *Hoxd13*, litters resulting from the breeding of XB mice expressed *Hoxd13*, although expression levels varied among the XB litters (Figure 5A). These results suggest that mice of the XB transgenic line expressed *Hoxd13* prematurely, before the 7-dpc stage, and the lethality observed in these mice was due to this premature expression.

Previously, we proposed that a repressive region outside of the *HoxD* complex is responsible for the early repression of genes in the *HoxD* complex, preventing the premature expression of *Hoxd* genes before the 7-dpc stage [14]. The presence of an extra copy of the XB fragment, which acts as a constitutive boundary element when the regulatory sequence (BB fragment) is absent and when it is inserted between the repressive region and *HoxD* complex, may interfere with the repression from the repressive region thereby causing *Hoxd* genes—in this case *Evx2* and *Hoxd13* genes—to be expressed prematurely (Figure 5B). Taken together, these findings suggest that the XB fragment possesses protection activity against repression as well as enhancer blocker activity.

### XB protects against repression from chromosomal environment

Exogenous genes introduced into a genome often become repressed over time by the chromosomal environment surrounding their insertion sites. Our observations indicated that insertion of the extra copy of XB fragment protects the neighboring promoters, *Evx2* or *Hoxd13* in this case, against repression (Figure 5), we examined whether this protection is also operative when the XB fragment is positioned randomly in the genome.

**Figure 6:**
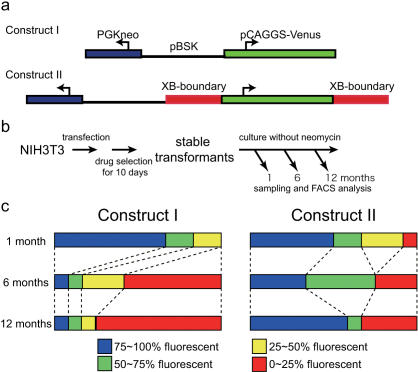
Position-effect blocker activity of the XB fragment. (A) Constructs introduced into the NIH3T3 cell line. Construct I (top) contains a CAGGS promoter-driven fluorescent protein (Venus) and a neomycin-resistance gene, enabling G418 selection of stable transformant colonies of transfected NIH3T3 cells. Construct II (bottom) is organized similarly, except it contains XB blocker fragments bordering both sides of the Venus expression marker gene. (B) Experimental scheme illustrating the transfection scenario. Stable transformants of both constructs were selected using G418. Colonies were maintained in culture for one year. We selected samples from each colony after 1, 6, and 12 months for FACS analyses. (C) Fluorescence assessment of 12 colonies harboring either Construct I or Construct II. Fewer fluorescent cells were observed in colonies lacking the XB fragment, while numerous fluorescent cells were observed in colonies harboring the XB fragment, indicating that the XB construct (Construct II) maintained Venus expression.

We made two constructs—one containing the fluorescent protein Venus (Construct I), and the other containing the Venus marker gene bounded on both sides by the XB fragment (Construct II) (Figure 6A; for XB, see Figure 2, 3). Both of these constructs also harbored a neomycin-resistance marker to facilitate isolation of stable transformants. These constructs were introduced into NIH3T3 cells, which were then cultured in G418-containing media for one week to select neomycin-resistant colonies of randomly integrated transformed cell lines (Figure 6B).

Twelve stably transformed colonies containing each construct were selected. Most of the clones contained few copies of the transgene (1–5 copies), as estimated by genomic Southern hybridization. After isolation, colonies were maintained in media lacking neomycin and maintenance of Venus fluorescence was assessed by fluorescence-activated cell sorting (FACS) (Figure 6B). After 1 month of culturing in media lacking neomycin, most clones continued to express the Venus marker, except for one clone harboring Construct II in which the transgene may have become disrupted at the beginning of the experiment (Figure 6C). After 6 months of culturing, Venus expressed by clones harboring Construct I were dramatically different from that by clones harboring Construct II (Figure 6C). Seven of 12 Construct I clones lost fluorescence almost completely, while only three of 12 Construct II clones lost fluorescence. After one year of culturing, nine of 12 Construct I clones lost fluorescence, whereas seven of 12 Construct II clones retained fluorescence nearly completely (>75% of cells retained fluorescence). Overall, the XB-flanked Venus marker apparently maintained fluorescence more efficiently than the unflanked Venus marker (Figure 6C). These results strongly suggest that the XB fragment conveys protection regardless of its position in the genome.

## Discussion

Mammalian *Hox* genes form a tightly packed gene cluster within the genome [24]. Their genomic structure is believed to be highly correlated to the transcription mechanisms underlying their collinear expression [25]. Recent studies have suggested that the initiation of *Hox* expression requires the DNA domains outside of the gene cluster, domains that regulate the collinear expression of *Hox* genes during development [14], [26], [27].

On the other hand, the tight packing structure of *Hox* genes makes it difficult to alter the expression profiles of these genes and to explain how each gene is influenced differently by a common regulator. Therefore, distinguishing the expression patterns of each *Hox* gene may require an enhancer insulator or boundary element system similar to that proposed in *Drosophila melanogaster* [2]. We have previously presented several lines of evidence that such regulatory elements exist in the mammalian *Hox* complex [9], [18], [28]. In the present study, we determined the functional sequence of one boundary element of the mouse *HoxD* complex and analyzed the mechanisms of its function.

### Tissue specificity of enhancer–blocker function

The expression profiles of *Evx2* and *Hoxd13* genes have many aspects in common, such as initiation timing and expression in the future digit domain. However, *Evx2* is strongly expressed in the CNS, while *Hoxd13* is not. Interestingly, both of the enhancers that drive these genes in digits and brain are located and oriented similarly in relation to the *HoxD* complex, i.e., about 250 kb beyond *Evx2* and *Hoxd13* [17].

Several lines of evidence indicate that these differences derive from the ability of enhancers to differentially associate with different promoters. In the present study, we demonstrated that the functional region underlying insulator activity in our system comprises two units—a 2.5 kb fragment having constitutive blocker activity and a functional sequence regulating tissue specificity of the insulator activity. The detailed mechanistic basis of the interaction between these two functional sequences remains to be clarified. However, our series of targeted transgene experiments clearly indicated that the blocker regulator interferes with blocker (insulator) sequence function in digits and genitalia, thus supporting our hypothesis that chromatin makeup affects tissue-specific dynamics. In addition, methylation assays revealed that, in limbs or body parts in which the insulator sequence does not function, a hypomethylated form of the insulator prevails. The regulator sequence may play a crucial role in regulating the methylation of the insulator sequence, and hence in regulating insulator function.

### Comparison with β-globin system

The HS4 fragment from the chicken β-globin locus is a well-known insulator sequence identified in vertebrates. Extensive examination of HS4 showed that this sequence has two separable activities—insulator activity and position-effect protection activity—each of which arises from two short and distinct DNA elements [29].

Unlike in the case of chick HS4, we were unable to dissect the functional insulator fragment apart from the 2.5 kb fragment, and thus were unable to isolate from the* Evx2-Hoxd13* intergenic fragment the short sequence responsible for insulator activity or position-effect protection activity. In the chick β-globin locus, a CTCF-binding sequence is required and sufficient to block enhancer-promoter interaction [19], and another sequence is required for position-effect protection [29]. The *H19/Igf2* imprinting region also has CTCF-binding sites that function as insulator sequences in the imprinting system [21], [22]. In our system, however, we were unable to identify a CTCF-binding sequence by *in silico* screening or by chromatin immunoprecipitation assays (ChIP) using an anti-CTCF antibody (Kondo, unpublished data). Together, these findings suggest that the mechanisms underlying boundary activity in *Evx2*-*HoxD* are different from those underlying boundary activity in the β-globin and *H19* systems [21], [22].

### Functioning mode of the chromatin boundary element

We observed transgene expression in the CNS, limbs, and genitalia of transgenic mice harboring constructs in which promoters were inserted into the genomic region between *Evx2* and CNS and/or limb enhancers (GCR) [14], [17], [28]. Genes located beyond *Hoxd13,* however, showed no expression in CNS. Thus, boundary elements probably define the range of a chromosomal domain in which transcription factors can search for a promoter.

Enhancer titration or competition can be a possible mechanism of the *Evx2*-*Hoxd13* insulator [18], [28]. It is generally believed that some spatially close promoters compete for enhancers [30]. Indeed, the XB fragment may contain the *Evx2* promoter. However, the XB fragment—the enhancer blocker (insulator)—did not show promoter activity when we randomly inserted it into the genome as a transgene (Kondo, unpublished data). Additionally, titration or competition appears to occur specifically against the neural enhancer not the limb enhancer, even though *Evx2* promoter activity is prominent in both of these tissues. Specificity depended on the presence of an additional sequence (BNs fragment) next to the XB fragment, suggesting that even if the XB fragment has promoter activity when it is inserted near *Evx2*, promoter activity is not a decisive factor dictating whether the sequence will function as a blocker.

In addition, enhancer titration cannot explain the position-effect protection activity we observed in our study system. Although these findings may not directly exclude the possibility, we believe that the enhancer insulation occurring in the *Evx2-Hoxd13* system is not driven entirely by promoter titration.

Differential expression of *Evx2* and *Hoxd13* in the CNS may also be due to uni-directional repression activity. In this scheme, the XB fragment recruits repression from only one side (side adjacent to the *Bam*HI restriction site) of the flanking sequences. The BNs fragment, on the other hand, disturbs this repression activity in the limbs and genital bud, and as a result, posterior *Hoxd* genes are repressed in the CNS. The unidirectional repression activity scenario, however, cannot explain why *Evx2* and *Hoxd13* were prematurely expressed in XB-targeted transgenic mice. Additionally, this scenario cannot account for our transfection assay results showing that the genes adjacent to the *Bam*HI side of the XB fragment maintained active transcription and the XB fragment showed protection activity against repression from the genomic environment where the transgenes were inserted. These phenomena contradict the unidirectional repression hypothesis.

Boundary elements are candidate sequences that divide chromosomal DNA into units, regulating promoter-enhancer interactions and the extent of chromatin remodeling [8], [31]. Our findings prompt a revision of this traditional definition of chromatin boundary sequences, one including protection activity against negative chromatin spreading in addition to enhancer blocker activity. We showed that the sequence identified in this series of experiments is a proper boundary sequence, since it had position-effect protection activity as well as insulator activity. According to Corces and colleagues, boundary sequences are important for the three-dimensional assembly of chromosomal DNA [31]; these sequences, therefore, appear to represent constitutive activity that forms the structural basis of genomic DNA.

Although we could not detect the CTCF association on this boundary region, seeking associating factors on this boundary region is one of the important aspects to reveal the mechanisms of boundary function. Previous studies on *Drosophila* boundary elements raised several candidates for associating factors, such as BEAF-32 [32], *Su(Hw), Mod(mdg4)* [33], *Pleiohomeiotic* (*Pho*) or *Trithorax-like* (*Trl* = GAGA factor) [34]. Unfortunately, mammalian homologues of most of these factors remain elusive. However, potential association of *Pho* and *Trl* suggests the involvement of *Polycomb* group genes (PcG) and *trithorax* group genes (trxG) in the boundary function in *Drosophila melanogaster*. In vertebrate systems, PcG or trxG factors may also be involved in the boundary function through histone modifications.

In the present study, we demonstrated that the *Evx2*-*HoxD* boundary element operates in tissue- and time-specific modes. This boundary element is composed of two functional fragments—a constitutive boundary element and a boundary regulator fragment—the latter of which gives tissue specificity to the constitutive boundary element. The constitutive boundary element can protect promoters from the repressive influence of chromosomal environments when it is inserted along with promoters. This finding also indicates that constitutive boundary elements can be useful tools for stably expressing exogenous promoters in eukaryotic cells.

## Materials and Methods

### Targeted transgene

A construct containing the *Hoxd9*/lacZ indicator transgene, which was inserted into a *Hin*dIII site just downstream of the *Evx2* gene, and a test fragment (from the 5′-flanking region of the transgene) was introduced into R1 ES cells by electroporation. ES cells were selected by using neomycin resistance, and homologous recombinants were isolated by genomic Southern hybridization as previously described [14]. Chimeras were created with these homologous recombinant ES cells to establish targeted transgenic animals.

### Cell culture

NIH3T3 cells were cultured in Dulbecco's Modified Eagle's Medium supplemented with 10% fetal bovine serum. Constructs containing either a Venus expression marker gene or a Venus marker flanked by candidate boundary sequences (XB fragment) were introduced into NIH3T3 cells by electroporation. Twelve colonies from each pool of transformants containing the two constructs were randomly isolated and used for further testing. We continued to culture these transformants, assessing their fluorescence 1, 6, and 12 months after starting the culture by FACS.

### Bisulfite DNA methylation assay

Genomic DNA samples were isolated from forelimbs and hindbrain regions of 11 dpc embryos. After digestion of DNA by *Xho*I and purification steps, digested DNA samples were subjected to bisulfite modification [23]. Modified DNA was amplified with PCR, then subcloned into pBSK for sequencing. We sequenced 10 colonies from each pool. The PCR primers we used were as follows:

Pair 1,

5′-GGGAGGATATAGGAAGAGTTG-3′,

5′-CTCTATACCCCCAATCAACAC-3′;

Pair 2,

5′-GTGTTGATTGGGGGTATAGAG-3′,

5′-ACCTCACCTCCCCTTACATTC-3′;

Pair 3,

5′-GTGTTAGGGGGTAAATTTTTG-3′,

5′-CAAAAATTCTCTTCTAATCCC-3′.

### Gene expression study

*β*-galactosidase staining and *in situ* hybridization were performed according to established protocols. The *Hoxd13* probe was described by Dollé et al. [35]. The *Evx2* probe was derived from an *Xba*I-*Bam*HI genomic fragment of the *Evx2* gene corresponding to the 3′ UTR of *Evx2* gene.

We also assessed *Hoxd13* gene expression by real-time PCR using a Corbett RG-3000 with Invitrogen Platinum SYBRGreen PCR kit. We isolated mRNA from 7 dpc embryos from three different pregnant wild-type mice (wild-type matings), six different pregnant BB/− mice (BB-targeted transgenic heterozygotic matings), and eight different pregnant XB/− mice (XB-target transgenic matings). Each cDNA sample was derived from mRNA pooled from each litter, which was composed of several embryos (e.g., three cDNA samples from wild-type mice; six cDNA samples from BB/− mice; eight cDNA samples from XB mice).

To control for the amount of cDNA, we carried out PCR with *β*-actin.

We used the following primers:

*β*-actin,

5′- CATGTTTGAGACCTTCAACAC-3′,

5′- GTGATGACCTGGCCGTCAGG-3′;

Hoxd13,

5′- GGTTTCCCGGTGGAGAAGTAC-3′,

5′- TGGACACCATGTCGATGTAGC-3′.

We performed PCR in triplicate for each pool of cDNA and calculated the amounts of products using a standard curve made by sequential dilutions of isolated cDNAs for β-actin and *Hoxd13*.

## Supporting Information

Figure S1 LacZ expression of targeted transgenic mice described previously [1]. (A) Positions of targeted transgene. The Hoxd9/lacZ marker transgene was inserted half-way between Evx2 and Hoxd13 by using the ES cell technique to produce relO mice. The Hoxd9/lacZ transgene is immediately downstream of Evx2 in relI mice. The resulting ES cells were injected into blastocysts to establish transgenic mice. (B) In relI embryos, the lacZ-staining pattern in the isthmus resembles the expression pattern of Evx2. (C) LacZ-staining pattern indicates that relO mice do not express the transgene in brain, which is consistent with our Hoxd13 in situ hybridization results.
(0.02 MB PDF)

Figure S2 Sequence of boundary fragment. (A) Physical map of region including boundary sequence. The boundary fragment is indicated in red line. The numbers in parenthesis indicate the distance from XhoI site in the first exon of Evx2. (B) Sequence of XB-boundary fragment. The initiation codon of Evx2 gene is indicated with blue font.
(0.02 MB PDF)

Figure S3 Sequence of boundary regulator fragment. (A) Physical map of region including boundary regulator sequence. The boundary regulatory fragment is indicated in dark blue line. The numbers in parenthesis indicate the distance from XhoI site in the first exon of Evx2. (B) Sequence of BNs-boundary regulator fragment.
(0.02 MB PDF)

Text S1 Supporting Information legends and references.
(0.03 MB DOC)
