# Supplementary figures and images for: Evx2-Hoxd13 Intergenic Region Restricts Enhancer Association to Hoxd13 Promoter
Source: PLoS One. 2007 Jan 24;2(1):e175. doi: 10.1371/journal.pone.0000175 (PMC1766471; doi:10.1371/journal.pone.0000175)

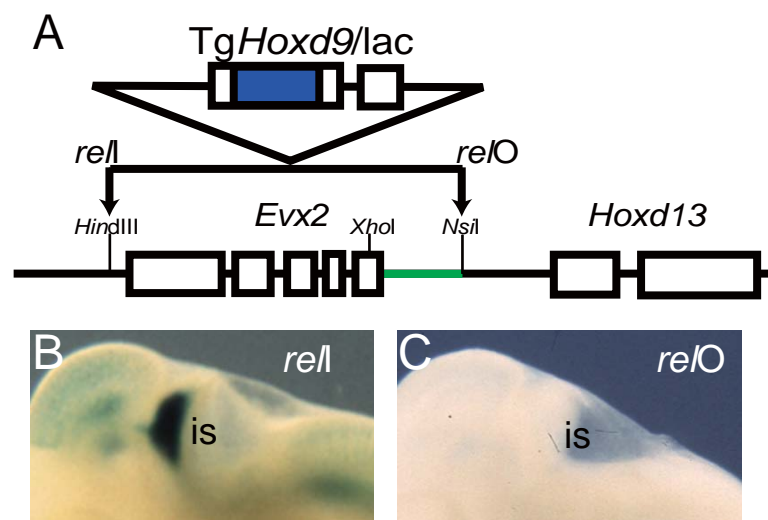

Supplement: Figure S1 — LacZ expression of targeted transgenic mice described previously [1]. (A) Positions of targeted transgene. The Hoxd9/lacZ marker transgene was inserted half-way between Evx2 and Hoxd13 by using the ES cell technique to produce relO mice. The Hoxd9/lacZ transgene is immediately downstream of Evx2 in relI mice. The resulting ES cells were injected into blastocysts to establish transgenic mice. (B) In relI embryos, the lacZ-staining pattern in the isthmus resembles the expression pattern of Evx2. (C) LacZ-staining pattern indicates that relO mice do not express the transgene in brain, which is consistent with our Hoxd13 in situ hybridization results. (0.02 MB PDF) [file pone.0000175.s001.pdf]
