# Supplementary material for: Evx2-Hoxd13 Intergenic Region Restricts Enhancer Association to Hoxd13 Promoter
Source: PLoS One. 2007 Jan 24;2(1):e175. doi: 10.1371/journal.pone.0000175 (PMC1766471; doi:10.1371/journal.pone.0000175)
Supplement: Figure S2 — Sequence of boundary fragment. (A) Physical map of region including boundary sequence. The boundary fragment is indicated in red line. The numbers in parenthesis indicate the distance from XhoI site in the first exon of Evx2. (B) Sequence of XB-boundary fragment. The initiation codon of Evx2 gene is indicated with blue font. (0.02 MB PDF) [file pone.0000175.s002.pdf]

Supplemental Figure s2, Kondo

A

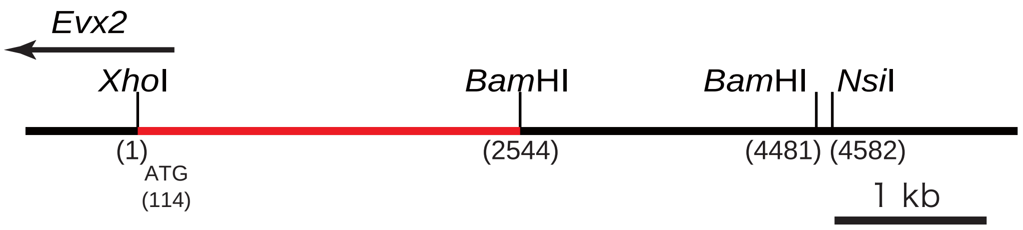

B

CTCGAGCACA GCACCGCCAG CTGAATCGGA CAAACTGGAG AACCTCTTGC CAGCAGTAGG GCTGTGGAGC CCTCTTTCCA TCAGAATCAT CTCTTTTCTT

ATTCTTTCCA T**CAT**CTCAGC TTTCTAAAAA ATGTCACAGT GGCCTGGCTG TCCCGTCTTA ATGATGGGCT GCGCCCAGGG CTGATTCTCA TTCAGCCCCA

Evx2 start codon

GCAAGCGGCT CTAAATATTA TTATCTCTTT TGGAGGGAGG CGGAAAAAAT TGTGGGATGC CAGAGCGAGG GAATGACTGG TCAAAATGAC CCATACATCC

TTCCGAGTCG GAGATGTCAT TCATCAAAAA GTAGCGCCCG CTCGTCATTA AGGTACGAAT GACGCCGTTC GAATAATCAT TTATTGTAAC AGGTTTATAA

GCAAATAAAT ACACGCTCCT GTCAGTGGGT AATGAAGGCG ACTTCAGAGC AGACAAATAG ATCCAGGTAG GAGACGAGAA GACACGAGAA GTGAGGAAGA

AGGCTCCGGT CCTTTGCCCG CTCCCAGCCA GTTCTGCTCG CCCGGGGGTT TGGCCTCGGG GGTGAAATTG ACAGTCTGAT TAATAGAGCA CGCCGCGGCG

CATTTCCCCC TTCATTTAGG GGCATCATTA CGCTCTCAGT TTGCTGGGTT GCCCCTTAGG TCCTAATCAT GCAGCGCCTT CCTCATTTCT CCACGAACAC

AGATACGTGT TAAATAATAG ATAATGCTTT GATTTTTCCA GGGTAGATTC TTGAGAAGTT TTTGTTTTGT TTTGTTTTGT TTTGTTTTGC CTTAACATTT

ACAAGCTGGG GGCGTAAGGG GGGCGGGTAA AGGGGAGGAT ACAGGAAGAG TTGCGTAAGA AGACCTCGCG CCCACTCCTG TCAGGATTAC TTATCTTTCT

GTTTTAAGAA CCAAATATTA ATAATGAGTA TTTTATTTAA CAAATAGAAT TCTGAGTTCT TTCTCTCTCC TTACTTTTTC CTCTCAGTCT TGATTCTCCC

CTCCAACACA CACACACACA CAGAGAGAGA GAGAGAGAGA GAGAGAGAGA GAGAGAGAGA GAGAGAGAGA ATATATCTTG TAGACTTTTG AAAATGCTTT

CTTGCTACGT GGCTGAGGAG GTACACAGGA CCCGTGCCGT GCCTCCAAGT GGAAATCATT ACATCTTCTC TTATTTTCTA CCACTGCTTG CAGGCTAACT

TCTGAACTCG AAGACCTTAG ATTTTTTGTT TTATAATCCT CTGATGGATA AGCTTCTCAC CCTCGCATTC TCCCTTAGGG GAAATAAACT AGACTGGAGG

ATCTGATAGT AGAAATTGAC AGCTAACTTT AAACACAATC ATGATTTACC GTACCATAGC TCCCCATCAC AGAACCCACT TTTTAAAATT GCCGGCTTTC

TCCCACCCTT CTCAAATAGA GGAATTCGTG GTGAAGTAAC TGGGTTTTGG GGGTCAAAGT GGAGGGAATG GAATCTCCTG CTGCCTCCCT TCCTTTCCCA

CTTTGTTCTT CTTCCACTTC TCCATCCTCC CCCTCCTCCA CCCTAGGCCC TTACCTGTGG CCCCCGGTGT TCCTGGTGCT GACTGGGGGC ACAGAGAGTC

TCCAACCAGC CTTGCCTCTT CTCCTTCCCG CCTGAGGCTG GAGGCTGAGC CTCTTGGCGA TAACTTTGAT AGTCATAATG ACGC**C**GGTGG CAACCATTTT

AAGGAAGATC AATCTTCACT CAAATG**C**GCT CATCTCCTCC CGCGCTTG**C**G **C**CCCACCCTG CC**C**GACTCAG ATGCGTCGTG CCAGTTTGCC **C**GCCTGCTGC

CCCAGAAGCA GAGCTTTGTC CGCACTGGCG AGGTCCACGG AAGACAGGGA CAGGACACAG TGGTCTAGCG CGTGAGCTTC **C**GAAGCAGCC TAGAGAGCCT

GGCAGTCTTG ATGCTGGTTT TGCATCC**C**GG TCACAGGACT AACTGAGACC CTAACC**C**GGG CCCCCCTCCT TCCCGCCTTG AGACACACCC AGCATATTGG

GGTGTGTTAG GGGGTAAACC TCTGATCTAA TTGCCCATCT GTCTGTTGG**C** GCTTTCTTTT TAACCTTGGA CCCGTTGGGG GCGCGTCAAG AATGTAAGGG

GAGGTGAGGT GTGAAATATA TTTATATACA TGTGTATACA TATATAGTCA ATGCCAAAAG TTTTTCACCT CTTCCCCTTA GCGCCAACTT ATTTCTACAG

GCAAGCTTGC TGGAAAAAAA AAAAAAAAAA AAAAAAAAAA CAATCCCACC TCTTTAAAGT TGTAAGTGGA AAATTGCATA GGCTCGCAGG CTTAGGAGAA

TTGTCTGGTG GG**C**GATGACC TCCATTTGAG AATTTGACCC AG**C**ATCCACC TTTGCCC**C**TG TCCCAGTGGC TATTCTACCC TAGTGGTCAT TCAGTTCCTG

GCACTTGCAT TGTCCTCAAG CAGGATTTTG GCTACCTCTC TTCGCTAAT**C** ACACGTCCAG CTCTGCGGGC GCCCCCAACT GCTTCTCAGC CAGGACGCCC

AGCTCCACCG GTCCAGGCAG TCTGGGCCCC AGTGTCTCTG GGAGGATCC
