# Supplementary material for: Evx2-Hoxd13 Intergenic Region Restricts Enhancer Association to Hoxd13 Promoter
Source: PLoS One. 2007 Jan 24;2(1):e175. doi: 10.1371/journal.pone.0000175 (PMC1766471; doi:10.1371/journal.pone.0000175)
Supplement: Figure S3 — Sequence of boundary regulator fragment. (A) Physical map of region including boundary regulator sequence. The boundary regulatory fragment is indicated in dark blue line. The numbers in parenthesis indicate the distance from XhoI site in the first exon of Evx2. (B) Sequence of BNs-boundary regulator fragment. (0.02 MB PDF) [file pone.0000175.s003.pdf]

A

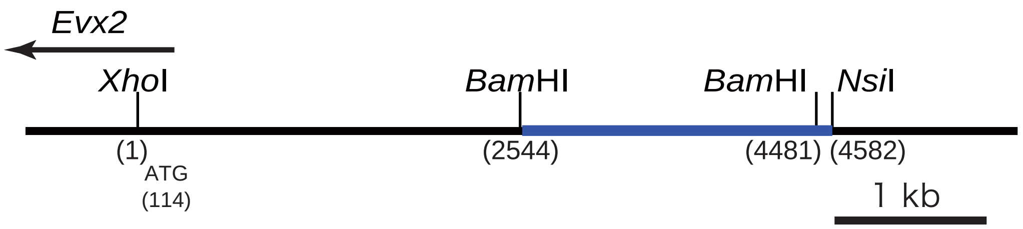

B

GGATCCTGCT ACCTAATCCT TCTCTACCCA AGGGACTAGA AGAGAATCTT TGGTCTAGGC TAGGCTCCAT GGGGGGCCAA GACCCTGGCA GCAAGTGGTC

GCTCAGCCTG GGCGGAAATC CGCCGATGCC TTTATTGCTG TCATTTGGCG CCCCCATCTG TTTTCCTCGC GGTTTCAGCA GCTGTGAGCT GCTTTTCTGT

CGCAGAAACC TCAGTGGCCT AGGAAGATTT CTTACCTTCT TCTACGACCC ACCGCCTCTG GTTCCAAAAG CTGGCCAAGT ACGACTCCAC TCTACTGAAA

ATGAAAAAAA AATCATTTCT CCGCGACACC TTCATTCATT AGTGGTTTTG AAACTACCTT GCACCTTTTA AGTAACACTG ATACTGCATT TCCAGGATGT

CTATTGCCCC ACATATACTT GTGATCTTGT CAGGGAAACT ACCTCAATGA AAAACATCCA TAACTTTTCA CTGCCCTTAG CAGAGGATAG CATTTCACAG

TGACTATTCC ATTTGAATAG TTAATGTTCC TTTAGAACAT TACCTATTAC TAGTAATAAT ATTGACTAAT TTGTATAACT CATTTAGTAT ACAATTACAA

GTGGCTTTTT ACATAGATCT ACCTTCTGAA GACCAGAGAA CCCCTGGGGA ATTGCCCCGC CCCTTTAAGG AAACCTCCTA CACAGAGAGC TTTGGTAATT

GTTCATGGTT TATACTTATC TCCAATAATG GATGTCATGG GGGGTTGAAA GTTTTGCATA ACCGCTTTTT TTTTTCTTCA TGTTACCTGT CTTATTTAAA

GGCAGGCCTA CCTCAAAAAC ATTACACCAG TGGAGGAGAG AGAGAGAGAG AGAGAGAGAG AGAGAGTTAC ATTTGTTGAA AAAATAGTCA TTTCATATCC

TTTCCAGAAA GGAGAGGATG AAATTAGAAA TGGACCCAGT TTTCAGTTTC TGATATCTTC AAAGTACCAT CACCAAGAAC AAGAACACTC AGACAAAAAT

CTAACCCAAA CCCCATGCCT TCAAAGGGCA TCTTCCACCT ATGCGAAGGG CATGCCAAAT TTTTAAGATT GGGAGTGAGG TGACATACAG GAAAAAATTT

CTCTGTATTA CCCAAAAAGA ACAGTTTTGC TGGCAAGAAT GATGTAAACA AAGCAAGGGC ATTTTCTTTT CCTCCTTTTC TTTTTCTCCT TCCTTCCTTT

CTTCCTTCCT TCCTTCCTTC CTTCCTTCCT TTCTTTCTTT CTTTCTTTCT TTCTTTCTTT CTTTCTTTCT CCTGGGGTGG GGTTAGACTG CCAAACTAAG

TATTTGTTTC TTGTAAACAG TGAACATTTG ACCTGTGACT TCAGGTTAAA ACTTGTTCTA GACTTGCAGA GGGATGGGTC AAGAGTAAAG CCCCGTCGAG

CAGCAACAGG GCCACGGGAT GCGGTGGTCT CACCCAGCTC TGGGCTTCCC GGTGGAAGAT GCCAGCCACC CGTTCTCAAC CCGAAGTAAG TCTCAAGCTC

GCTGGAGAAC TTATGGGAGT GCAGATGTTG TGAACGGGCA GTAAACACCT GCAGAGTCTG AGCTGCGGGC AGGGCATGTT TTCTTTAGGC TAAGTGTGGT

CTTCAGGACC AGGTTTCGAT TTATAAGAGA GGGTGAGGGT TGGACTAACC AAGCCAGTCT TCTAGGCTTC CTACTCTCAT TTCTCCTCAG GCAAGCCTCT

AGCTTTCTGC CTCGCCTGGG TCAGCTCATT TAATTTAGTT TTCCTTCTCC CCTCCACCTT CAACTCCAAG TTACCCTAGA ATGGTTGTAA GTGAGGGAAT

TGGTGGCTAC TTTCTTCCTG GACCCCGAGG CTAATCAGAT TTGCCAACTG CCAGGAGTTT AATGTACCCT CAGATCAGAC CCAAGAACTT GCAGGGACAT

CTAGCGCCAA GCGCGGGGAA CTGCGGTCGC CTTTCAGGGA TCCGGAGCAT ACCACTGGCA TCCTTTGCCA GTCTCTCAAC TCCTTCTACA TTTTTAGGCA

TTTTCCTACC CTTCACCCGC TTTGCTTCGC TGCTATGCAT
