# Supplementary material for: Evx2-Hoxd13 Intergenic Region Restricts Enhancer Association to Hoxd13 Promoter
Source: PLoS One. 2007 Jan 24;2(1):e175. doi: 10.1371/journal.pone.0000175 (PMC1766471; doi:10.1371/journal.pone.0000175)
Supplement: Text S1 — Supporting Information legends and references. (0.03 MB DOC) [file pone.0000175.s004.doc]

**Supplementary Information**

**Supplemental Figure Legend**

**Supplemental Figure s1,** LacZ expression of targeted transgenic mice described previously [1]. **(A)** Positions of targeted transgene. The *Hoxd9*/lacZ marker transgene was inserted half-way between *Evx2* and *Hoxd13* by using the ES cell technique to produce *rel*O mice. The *Hoxd9*/lacZ transgene is immediately downstream of *Evx2* in *rel*I mice. The resulting ES cells were injected into blastocysts to establish transgenic mice. **(B)** In *rel*I embryos, the lacZ-staining pattern in the isthmus resembles the expression pattern of *Evx2*. **(C)** LacZ-staining pattern indicates that *rel*O mice do not express the transgene in brain, which is consistent with our *Hoxd13* *in situ* hybridization results.

**Supplemental Figure s2,** Sequence of boundary fragment. (**A**) Physical map of region including boundary sequence. The boundary fragment is indicated in red line. (**B**) Sequence of XB-boundary fragment. The initiation codon of *Evx2* gene is indicated with blue font.

**Supplemental Figure s3,** Sequence of boundary regulator fragment. (**A**) Physical map of region including boundary regulator sequence. The boundary regulatory fragment is indicated in dark blue line. (**B**) Sequence of BNs-boundary regulator fragment.

# Reference

1. Kondo T, Duboule D (1999) Breaking colinearity in the mouse *HoxD* complex. *Cell* **97**, 407-417.
